# MORE THAN JUST A RIDE: TRANSPORTATION PROGRAMS TO MEET THE NEEDS OF OLDER ADULTS IN RURAL COMMUNITIES

**DOI:** 10.1093/geroni/igae098.0417

**Published:** 2024-12-31

**Authors:** Alycia Bayne, Alexa Siegfried, Skyla Chitwood, Nathaly Valdivia

**Affiliations:** NORC at the University of Chicago, Chicago, Illinois, United States; NORC at the University of Chicago, Chicago, Illinois, United States; NORC at the University of Chicago, Chicago, Illinois, United States; NORC at the University of Chicago, Chicago, Illinois, United States

## Abstract

Most older adults (age 65+) living in rural areas rely on personal vehicles for transportation. However, transportation is a significant challenge for older adults who need alternative forms of transportation, including those who do not drive or lack access to a vehicle. Fixed route transportation is limited in rural communities and may not meet the needs of older adults, particularly those who require assistance. Transportation barriers affect older adults’ health, mobility, independence, and quality of life. Recognizing these challenges, the Health Resources and Services Administration Federal Office of Rural Health Policy funded the NORC Walsh Center for Rural Health Analysis and the Rural Health Information Hub to develop the Rural Transportation Toolkit. The toolkit includes 15 promising program models to increase access to transportation, overcome barriers, and improve safety. To develop the toolkit, NORC conducted an environmental scan to identify programs and resources and interviewed representatives from nine rural transportation programs and four experts in rural transportation. The toolkit was first released in 2019, updated in 2024, and has had over 50,000 page views. This session describes toolkit findings, including transportation needs and challenges for rural older adults, and features program models practitioners can adapt. Program models include the Ride Sharing Model, Volunteer Driver Model, Voucher Model, and Coordinated Services Model. This session also describes how technology can improve rural transportation for older adults. This toolkit has the potential to help rural communities meet the transportation needs of older adults.

